# Cognitive function and associations with demographic, socioeconomic, health and behavioural factors among older adult men and women in rural Bangladesh: a population-based cross-sectional study

**DOI:** 10.1016/j.lansea.2025.100575

**Published:** 2025-04-07

**Authors:** Edward Fottrell, Harry Costello, Naveed Ahmed, Carina King, Sanjit Kumer Shaha, Tasmin Nahar, Malini Pires, Andrew Copas, Hassan Haghparast-Bidgoli, Joanna Morrison, Abdul Kuddus, Kishwar Azad

**Affiliations:** a UCL Institute for Global Health, University College London, London, UK; b UCL Institute of Cognitive Neuroscience, University College London, London, UK; c Centre for Health Research and Implementation, Diabetic Association of Bangladesh, Dhaka, Bangladesh; d Department of Internal Medicine, BIRDEM General Hospital, Dhaka, Bangladesh; e Department of Global Public Health, Karolinska Institutet, Stockholm, Sweden

**Keywords:** Cognition, Ageing, Bangladesh, Hypertension, Diabetes, Depression, Anxiety, Physical activity, Epidemiology

## Abstract

**Background:**

Cognitive impairment has a major impact on health, quality of life and survival and its increasing burden presents a critical global health challenge. Empirical population-based studies of cognitive function and its association with demographic, socioeconomic, health and behavioural factors among older adults in low-resource setting are rare. This study describes the burden of cognitive impairment and associations with demographic, health and behavioural factors among older adults in rural Bangladesh.

**Methods:**

We conducted a population-based cross-sectional study of a random sample of men and women aged 60 years and above in 96 rural villages in Bangladesh. Cognitive function was measured using the Bangla Adaptation of the Mini-mental State Examination (BAMSE), where higher score indicates higher function. Blood pressure, height and weight were measured using standard protocols and fasting glucose and 2-h oral glucose test were used to identify diabetes risk. Interviewer administered survey questionnaires assessed depressive symptoms, anxiety and self-reported health behaviours. Analyses were carried out separately for men and women and examined associations between sociodemographic, health and behaviour factors with BAMSE scores using robust Poisson regression.

**Findings:**

Data were gathered from 403 (216 female, 187 male) eligible participants. More than 50% of the population had at least mild cognitive impairment and women had lower cognitive scores than men. Younger age, higher education, wealth, and literacy were significantly associated with higher BAMSE scores among women and men. Associations with marital status varied between men and women, with being married having a positive association with BAMSE among women, i.e. higher cognitive function (relative score ratio (95% CI) 1.08 (1.02, 1.15), p = 0.013), but no association among men (0.94 (0.87, 1.02), p = 0.13). No clear associations were observed with diabetes or hypertension, but overweight and obesity were associated with an increased BAMSE score among women (1.10 (1.02, 1.19), p = 0.011) but not men (1.01 (0.94, 1.10), p = 0.70). Moderate and severe depressive symptoms were associated with lower BAMSE scores among men (0.90 (0.82, 0.99), p = 0.037), but not women (0.94 (0.83, 1.06), p = 0.31). Physical activity was associated with a relative increase in BAMSE score (1.08 (1.01, 1.16), p = 0.020) among women, though no association was seen in men (1.01 (0.95, 1.07), p = 0.76). The consumption of betel was associated with lower BAMSE among women (0.94 (0.89, 1.00), p = 0.056), but there was no evidence of association among men (1.01 (0.96, 1.07), p = 0.61).

**Interpretation:**

A large proportion of older adults in rural Bangladesh have impaired cognitive function and there are important gender differences in the distribution of cognitive scores and their association with demographic, socioeconomic, health and behavioural factors in this population. Improvement in health and social care systems, taking into account the specific social, economic and gender dimensions of the context, are needed to prevent and manage the burden of cognitive decline in rural Bangladesh.

**Funding:**

This survey and analysis were part of the Bangladesh Diabetes Community-Led Awareness, Response and Evaluation (DClare) study funded by 10.13039/100014013UKRI/10.13039/501100000265MRC (MR/T023562/1) under the Global Alliance for Chronic Diseases Scale-Up Programme.

**Research in context**

**Evidence before this study**

Cognitive impairment is a growing global health concern, particularly in low- and middle-income countries (LMICs), where dementia prevalence is rising rapidly due to population ageing and higher frequency of potentially modifiable risk factors. Understanding the burden of cognitive impairment and its associations with health and modifiable risk factors is essential, yet there is a lack of population-based data on cognitive function and health and behaviour measures among older adults in LMICs. We searched PubMed (from inception to May 2024) for studies on cognition and modifiable risk factors in lower middle-income countries, using the terms “cognitive impairment,” “aging,” “low income,” “Bangladesh,” and “modifiable risk factors.” Few population-based studies in LMICs were identified, with most research relying on hospital-based or small urban community samples, limiting generalisability. There is a critical need for large-scale data on cognitive function and its determinants in these settings.

**Added value of this study**

We conducted a large population-based study to estimate the prevalence of cognitive impairment in rural Bangladesh and examine its associations with socio-demographic, economic, and health factors. Over 50% of adults aged ≥60 years have at least mild cognitive impairment. We identified important gender disparities with women exhibiting lower cognitive function scores and distinct associations between socio-demographic and health variables and cognitive function.

**Implications of all the available evidence**

Two thirds of people with dementia worldwide live in LMICs, with this number projected to rise more rapidly than in higher-income countries. Our findings emphasise the need for accessible cognitive health strategies in LMICs, including developing health and social care systems with clear diagnostic, treatment, and support strategies for individuals with cognitive impairment and their caregivers. Such efforts must be tailored to gender specific needs, including access to education and physical activity for women and reducing social isolation.

## Introduction

Cognitive impairment—where an individual has difficulty with thinking, reasoning, memory or attention^1^—is a major concern for older adults and their families and has significant economic, social and health impacts. Lower cognitive function is associated with increased risk of mortality, disability and poor quality of life.^2^, ^3^, ^4^ Due to demographic, epidemiologic and socio-economic transitions, the prevalence and impacts of cognitive impairment are certain to increase and therefore present a critical global health challenge.

It is estimated that 60% of people living with dementia—the loss of cognitive functioning to an extent that impacts on daily life and activity^5^—lived in low- and middle-income settings in 2020 and this will rise to 71% by 2050.^6^ However, there is a general lack of population-based data on cognitive function among older adults in low- and middle-income settings, including in Bangladesh where the prevalence of dementia is estimated to rise to 3.4 million cases by 2051.^7^ Whilst effective treatments for cognitive impairment remain elusive and weak health systems hinder diagnosis and care for people affected, it is important to understand sociodemographic, health and behavioural factors associated with cognitive decline to target interventions and effectively plan services.

Estimates of dementia prevalence and cognitive impairment vary for Bangladesh, depending on methods used, study location and timing. A community-based study conducted among adults aged 60 years and older in rural Matlab in 2003–2004 reported a dementia prevalence of 15.1%.^8^ A population-based survey conducted in Dhaka in 2014 reported that approximately 28% of adults aged 60 years and above had mild, moderate or severe cognitive impairment.^9^ Despite a high degree of missing data on cognitive function and a small sample size, the Dhaka study indicated that age, physical disability and social engagement were associated with cognitive impairment in adjusted analyses. A small study conducted in a specialist tertiary mental health facility in Dhaka in 2014 and 2015 reported lower levels of mild, moderate or severe cognitive impairment at 16.6%.^10^ A national cross-sectional study across seven administrative divisions in Bangladesh in 2019 reported 8% dementia using the Mini-mental State Examination (MMSE) and a cut-off threshold for 24, with notable variations by age, sex, education, marital status, occupation and division.^7^ Despite existing evidence of cognitive impairment in Bangladesh, specific diagnostic, treatment and care plans are lacking and a research gap remains to describe the burden and distribution of cognitive impairment alongside measures of physical health and modifiable non-communicable disease risk behaviours in rural, population-based samples. Therefore, we aimed to describe both the burden of cognitive impairment and identify associations with demographic, health and behavioural factors in a rural setting in Bangladesh.

## Methods

### Setting

In August–September 2021, as part of a follow-up to the 2016–2018 DMagic cluster randomised trial (trial registration ISRCTN41083256),^11^, ^12^, ^13^ we conducted a population-based cross-sectional study to describe cognitive function among older men and women living in 96 villages across four upazilas (sub-districts—Boalmari, Saltha, Madhukhali and Nagarkanda) in Faridpur District, south-central Bangladesh. These villages were purposefully selected in 2016 based on the 2011 Bangladesh Census,^14^ given their estimated population sizes between 750 and 2500 (i.e. medium sized villages), non-contiguity between villages (to avoid contamination in the DMagic trial), and accessibility to the district headquarters of the Faridpur Diabetic Association in Faridpur City.

### Sample

Using the household sampling frame developed for the DMagic endline survey in 2017/18, a sample of 20 adults aged ≥30 years and permanently residing in the study villages were randomly selected from each study village (total n = 1920) in 2021. First, twenty households with at least one eligible adult were selected using simple random sampling in each village, and then a single eligible adult from each household was selected using simple random sampling. This sample was intended to assess five-year post-randomisation effects of the DMagic interventions in the adult population aged 30 years and above. Sample size was based on power calculations related to the DMagic trial primary outcomes of diabetes and hyperglycaemia and full details have been previously published.^11^, ^12^, ^13^ The current analysis is restricted to individuals aged 60 years and above at the time of data capture, n = 403.

### Variables

Our outcome of interest in this study was cognitive function. We used the Bangla Adaptation of the Mini-mental State Examination (BAMSE) to assess cognitive abilities in our sample.^15^ BAMSE is based on the widely used Mini-mental State Examination (MMSE) tool,^16^ which is a brief instrument for cognitive assessment that requires literacy and has been applied in a number of different population groups.^17^ The BAMSE was adapted to be applicable for illiterate individuals and to be culturally relevant in Bangladesh and has been shown to be a useful method to assess cognitive function in this context.^9^^,^^15^ As with the MMSE, the BAMSE was not intended to provide a diagnosis of any particular classification of disease but rather to estimate the severity of cognitive impairment. It contains 12 items concerning orientation in time and place, repetition, concentration, memory, language and praxis. The possible values of BAMSE are 1–30, where a higher score indicates better cognitive function.

We also recorded data on study participants’ socio-demographic characteristics, including age, sex, education, religion, marital status, occupation, literacy, household assets, and housing characteristics using a structured survey instrument adapted from the WHO Stepwise tool^18^ and the 2014 Bangladesh Demographic and Health Survey.^19^ Literacy was assessed by asking respondents who claimed to be able to read and write to read aloud from a text card provided by data collectors. The survey instrument also recorded data on self-reported health behaviours of tobacco consumption, physical activity and the consumption of betel nut.

Blood glucose was measured using the One Touch Verioflex Glucometer (Lifescan,Inc.,Milpitas, CA 95035) in whole blood obtained by finger prick from capillaries in the middle or ring finger after an overnight fast. All individuals then received an oral glucose tolerance test whereby a 75 g glucose load was dissolved in approximately 250 ml of water and a capillary blood glucose measure was taken within 5 min of 120 min post ingestion to determine glucose tolerance status and differentiate between individuals with intermediate hyperglycaemia and those with diabetes according to WHO criteria.^20^ Individuals who reported a prior medical diagnosis of diabetes were not required to provide fasting and 2-h blood glucose measures so provided either a fasting blood glucose sample or a random blood glucose sample. Although capillary blood glucose concentrations may overestimate blood glucose concentrations compared to venous samples, the method is feasible and acceptable for epidemiological studies.

Blood pressure was measured using the OMRON HBP 1100 Professional Blood Pressure Monitor (Kyoto, Japan). Two measurements were taken at approximately 5-min intervals and the respondent's blood pressure obtained by averaging these measurements. Measurements of height and weight were taken with light clothes without shoes and using weighing scales that were calibrated daily by known weight.

We used the Patient Health Questionnaire 9 (PHQ-9), which is a nine-item questionnaire designed to screen for depression and has been used previously and validated in Bangladesh.^21^, ^22^, ^23^ All participants were screened using the two-item PHQ-2 tool and those who screened positive for possible depressive disorder (a score of 3 or more) completed the full PHQ-9 survey. Anxiety was assessed using the Generalised Anxiety Disorder Assessment (GAD-7), a scale developed to identify probable cases of generalised anxiety and to assess symptom severity, which has previously been validated in Bangladesh.^24^^,^^25^

### Procedures

Recruitment and training of data collectors took place in July 2021 and data collection took place between August and September 2021. Sampled individuals were visited at their household, informed of the study and consent was obtained. All sampled individuals in a single cluster were informed of the physical measurement requirements of the study and were requested to attend a local centre on the morning of a specified day following an overnight fast. The centre was established by the field team for the purposes of the study and was at a central, convenient location in the village. Collection of questionnaire data, including BAMSE, PHQ, GAD-7 and health behaviours took place at a private outside location near the respondent's home before or after the physical measurements or at the time of physical measurement in the testing centre.

Data were collected by 12 teams of fieldworkers comprising a total of 28 men and women with at least secondary education who were recruited locally and selected through a written assessment and interview. All fieldworkers underwent 10 days training on survey methods and how to take physical measurements followed by one-week supervised field practice and daily debriefs in villages in Faridpur that were not included in the study. Data collectors were supervised by four field supervisors with experience in survey methods. Each supervisor was responsible for three data collection teams, spending half a day observing and verifying data within each team at least every two days. Within each village, teams were aided by a village assistant who received a daily payment to coordinate study participants and assist data collectors in their duties. Questionnaire data were gathered using Samsung Galaxy Grand Prime large screen smartphones using ODK Collect. All survey procedures were conducted in line with COVID-19 safety precautions, including the use of face masks, and were in line with Government of Bangladesh guidance at the time.

Data were transferred from each data collectors’ smartphone onto a laptop in the field every two days by one of the field supervisors and gathered data were transferred from the laptop to the data manager in Dhaka once per week. Detected errors or requests for verification were sent back to the field team in Faridpur.

### Analysis

Distribution of BAMSE scores was described for the whole study population and separately for men and women using a histogram and by category of severe (≤9), moderate (10–20), mild (21–24) or no (25–30) cognitive impairment using the cut-offs applied by Hossain et al. (2023).^9^ As with the MMSE, the BAMSE is negatively skewed in a general population, with a large number of participants scoring the maximum value of 30. We dealt with this skewness by applying a generalised linear model, namely, Poisson regression with log link function.

Education recorded as the highest level of education completed was categorised into three groups (none, primary, secondary or higher). Data on household asset ownership and housing characteristics were used to derive a wealth index using Principal Components Analysis (PCA) among the full survey sample (i.e. aged 30 years and above) following methods recommended by Demographic and Health Surveys.^26^ All assets and housing characteristics included in PCA were either binary (asset ownerships) or, if categorical (housing characteristics), were converted to binary variables before including in PCA. We used correlation matrix and Cronbach's Alpha to check internal consistency of the items included and checked the distribution of the scores for the first PCA component, confirming that they fall within the acceptable boundaries of a normal distribution. The PCA-derived wealth index was then grouped into tertials. The range of paid employment responses recorded were grouped into three broad categories (none, manual labour (including farming), and professional/clerical occupations). BMI (weight divided by height squared) was analysed as a continuous variable and categorised as overweight and obesity in accordance with recommended cutoffs for South Asian populations as BMI greater than or equal to 23. Categorisations of severity of anxiety were none/mild (<10) and moderate/severe (10–21) based on established cut-offs using GAD-7 scores.^24^ Severity of depressive symptoms was categorised as none/mild (<10) and moderate/severe (10–27) using previously applied cut-offs.^27^ Hypertension was defined as mean systolic blood pressure (SBP) ≥ 140 mmHg or mean diastolic blood pressure (DBP) ≥ 90 mmHg, or current treatment with antihypertensive medication. Individual's diabetic status was also recorded based on WHO definitions and blood glucose cut-offs or a prior diagnosis of diabetes by a medical professional.^20^ Self-reported health behaviours of current daily use of tobacco products, current daily consumption of betel and a minimum of 150 min of physical activity per week (the WHO cut-off for minimal levels of physical activity) were treated as dichotomous exposure variables.

Considering the marked differences in gender norms and practices related to health and behaviours in the study setting,^28^^,^^29^ all analyses were performed separately for males and females. BAMSE score was summarised for each exposure. Summary statistics and BAMSE scores were adjusted for the cluster design and weighted to account for unequal probability sampling of a fixed number of households within villages and a single individual within households using Stata's ‘svy’ command. Sampling weights were based on selection in the main survey, i.e. the inverse probability of selection among the eligible population aged 30 years and above. Robust Poisson regression accounting for weights and cluster design was used to provide crude estimates of associations between demographic, socioeconomic, health and behavioural variables and BAMSE score, presented as score ratios i.e. the relative change in BAMSE score.

Selection of variables for adjustment in multivariate analysis was theory driven, based on experience, subject knowledge and contextual understanding, as well as consideration of statistical associations between sociodemographic parameters and BAMSE outcome. Considering inter-relationships between sociodemographic factors and to avoid inclusion of highly correlated variables in a model, age and wealth were selected for inclusion in multivariate analyses because of their social and statistical correlation with education, literacy, occupation and religion. For analysis of associations between health and behaviour variables and BAMSE we also included adjustment for DMagic trial arm allocation as this is plausibly associated with exposure and outcome measures. Predictive margins were estimated for each exposure variable on BAMSE score based on regression models and the means and 95% confidence intervals were plotted to visualise associations by sex.

All analyses were done using STATA/MP version 18.5.

### Ethics

Written informed consent was obtained from all participants before data collection, or a thumb print for those unable to write. Ethical approvals for the DMagic trial and for this follow-up study were given by the University College London Research Ethics Committee (ref: 4766/002 and ref: 4199/007) and the Ethical Review Committee of the Diabetic Association of Bangladesh (ref: BADAS-ERC/EC/t5100246 and ref: BADAS-ERC/E/19/00276).

### Role of the funding source

The funder had no role in study design, data collection, data analysis, data interpretation, or writing of the report.

## Results

**Fig. 1:**
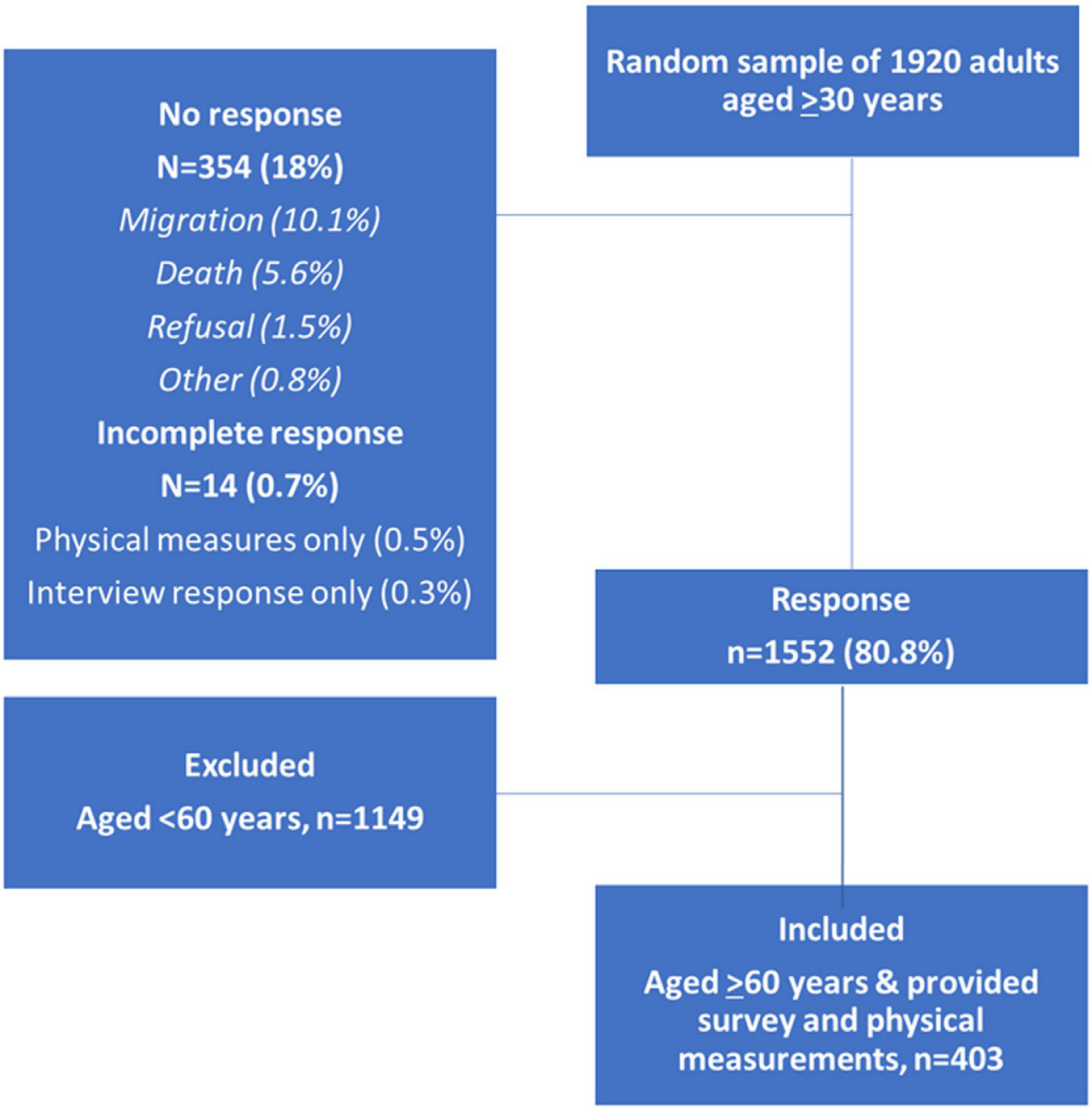
**Study sample and response rates**.

Of the 1920 sampled individuals, 1552 (80.8%) participated in the interview survey and physical measurements (Fig. 1). Response rate was higher among females than males (85.2% vs. 76.3%, p < 0.0001) and responders were younger than non-responders (52 years vs. 56 years, p < 0.0001). 403 respondents across 93 study villages were ≥60 years and therefore eligible for this sub-study, a mean of 4.3 (sd 1.8) per village. There was no evidence of a sex bias in responders aged 60 years or above in the sampling frame (77.8% female vs. 72.2% male, p = 0.13), but responders in this age category were younger than non-responders (70 years vs. 74 years, p < 0.0001).

Study population characteristics are summarised separately for men and women in Table 1. Age ranged from 60 to 96 years, with a mean age of the sample of 70.3 years (sd 7.5) for females and 70.9 (6.9) for males. The majority of female participants had no formal education (81.5%), were unable to read or write (86.7%), and not currently engaged in paid employment (98.3%). Among male respondents, 61.6% had no formal education, 71.0% were illiterate, and less than one third (31.5%) had no paid employment, with the majority (58.1%) engaged in manual labour. Whilst more than 91% of men were married, 54.8% of women in the sample were unmarried, with the vast majority (96.7%) of currently unmarried women being widows. The majority of male and female participants were Muslim.

**Table 1:** BAMSE score distributions and associations with socio-demographic and health parameters among adults aged 60 years and above in rural Faridpur, Bangladesh.

	Female				Male			
	N (weighted %) or mean (SD)	Median BAMSE score (IQR)	BAMSE score ratio (95% CI); p-value		N (weighted %) or mean (SD)	Median BAMSE score (IQR)	BAMSE score ratio (95% CI); p-value	
			Crude	Adjusted^c^			Crude	Adjusted^c^
Total	216 (100%)	22 (18–25)			187 (100)	26 (24–29)		
Age, years (mean (SD))	70.3 (7.5)		0.99 (0.98, 0.99); p < 0.0001	0.99 (0.98, 0.99); p < 0.0001	70.9 (6.9)		0.99 (0.99, 1.00); p = 0.0063	0.99 (0.99, 1.00); p = 0.005
Education								
None	174 (81.5%)	21 (18–24)	Ref	Ref	111 (61.6%)	26 (22–28)	Ref	Ref
Primary	26 (10.1%)	26 (22–29)	1.23 (1.14, 1.33); p < 0.0001	1.17 (1.09, 1.27); p < 0.0001	32 (16.0%)	28 (25–30)	1.10 (1.04, 1.16); p = 0.0005	1.08 (1.03, 1.15); p = 0.0033
Secondary or higher	16 (8.4%)	29 (26–29)	1.29 (1.16, 1.43); p < 0.0001	1.19 (1.09, 1.30); p = 0.0002	44 (22.4%)	30 (28–30)	1.17 (1.11, 1.23); p < 0.0001	1.13 (1.07, 1.19); p < 0.0001
Currently married								
No	133 (54.8%)^a^	20 (17–23)	Ref	Ref	16 (8.6%)^b^	27 (24–30)	Ref	Ref
Yes	83 (45.2%)	23 (20–28)	1.15 (1.08, 1.23); p < 0.0001	1.08 (1.02, 1.15); p = 0.013	171 (91.4%)	26 (24–29)	0.99 (0.90, 1.09); p = 0.89	0.94 (0.87, 1.02); p = 0.13
Wealth tertile								
Most poor	84 (36.4%)	20 (17–23)	Ref	Ref	69 (40.0%)	25 (22–28)	Ref	Ref
Poor	60 (27.5%)	22 (18–26)	1.08 (0.98, 1.19); p = 0.11	1.07 (0.98, 1.17); p = 0.11	56 (30.3%)	27 (24–29)	1.07 (0.98, 1.16); p = 0.13	1.06 (0.98, 1.14); p = 0.17
Least poor	72 (36.2%)	22 (19–29)	1.15 (1.06, 1.25); p = 0.0008	1.15 (1.05, 1.25); p = 0.0014	62 (30.7%)	29 (26–30)	1.14 (1.07, 1.22); p = 0.0001	1.14 (1.07, 1.22); p = 0.0001
Literate								
No	187 (86.7%)	21 (18–24)	Ref	Ref	131 (71.0%)	26 (23–28)	Ref	Ref
Yes	29 (13.3%)	29 (27–30)	1.33 (1.23, 1.42); p < 0.0001	1.25 (1.18, 1.34); p < 0.0001	56 (29.0%)	29 (27–30)	1.16 (1.11, 1.21); p < 0.0001	1.13 (1.08, 1.18); p < 0.0001
Religion								
Other	19 (11.6%)	26 (18–30)	Ref	Ref	17 (8.9%)	29 (28–30)	Ref	Ref
Islam	197 (88.4%)	21 (18–24)	0.89 (0.78, 1.00); p = 0.057	0.92 (0.82, 1.02); p = 0.12	170 (91.1%)	26 (23–29)	0.90 (0.84, 0.96); p = 0.0008	0.93 (0.88, 0.98); p = 0.0058
Paid employment								
None	211 (98.3%)	22 (18–25)	Ref	Ref	62 (31.5%)	25 (22–29)	Ref	Ref
Manual labour	3 (1.1%)	17 (17–17)	0.81 (0.76, 0.86); p < 0.0001	0.95 (0.82, 1.10); p = 0.48	103 (58.1%)	26 (24–29)	1.07 (0.98, 1.16); p = 0.11	1.04 (0.97, 1.12); p = 0.27
Professional/clerical	2 (0.6%)	27 (18–17)	1.13 (0.91, 1.40); p = 0.26	1.40 (1.09, 1.80); p = 0.0082	22 (10.4%)	29 (25–30)	1.06 (0.90, 1.24); p = 0.49	1.01 (0.87, 1.16); p = 0.91
DMagic intervention exposure^d^								
Control	74 (34.3%)	22 (17–25)	Ref	Ref	52 (29.5%)	28 (25–29)	Ref	Ref
mHealth	80 (36.5%)	20 (17–24)	0.98 (0.90, 1.06); p = 0.60	0.98 (0.90, 1.06); p = 0.57	60 (28.5%)	26 (21–29)	0.87 (0.80, 0.96); p = 0.0040	0.89 (0.82, 0.96); p = 0.0022
PLA	62 (29.2%)	22 (20–27)	1.10 (1.02, 1.20); p = 0.020	1.10 (1.02, 1.18); p = 0.0097	75 (42.0%)	27 (24–29)	0.96 (0.91, 1.02); p = 0.19	0.96 (0.91, 1.01); p = 0.14

a 126 (96.7%) widowed, 7 (3.3%) separated or divorced.

b 13 (77.9%) widowed, 1 (2.3%) separated or divorced, 2 (19.9%) never married.

c Education, marital status, literacy, religion, occupation and DMagic intervention exposure adjusted for age and wealth tertile. Age adjusted for wealth tertile. Wealth tertile adjusted for age.

d Full intervention details and impact are described elsewhere.^12^^,^^30^, ^31^, ^32^ mHealth intervention consisted of twice-weekly health promotion voice messages delivered to mobile phones in intervention villages over 16 months; PLA—Participatory Learning and Action consisted of monthly group meetings to facilitate community action in addressing diabetes risk in their community.

**Fig. 2:**
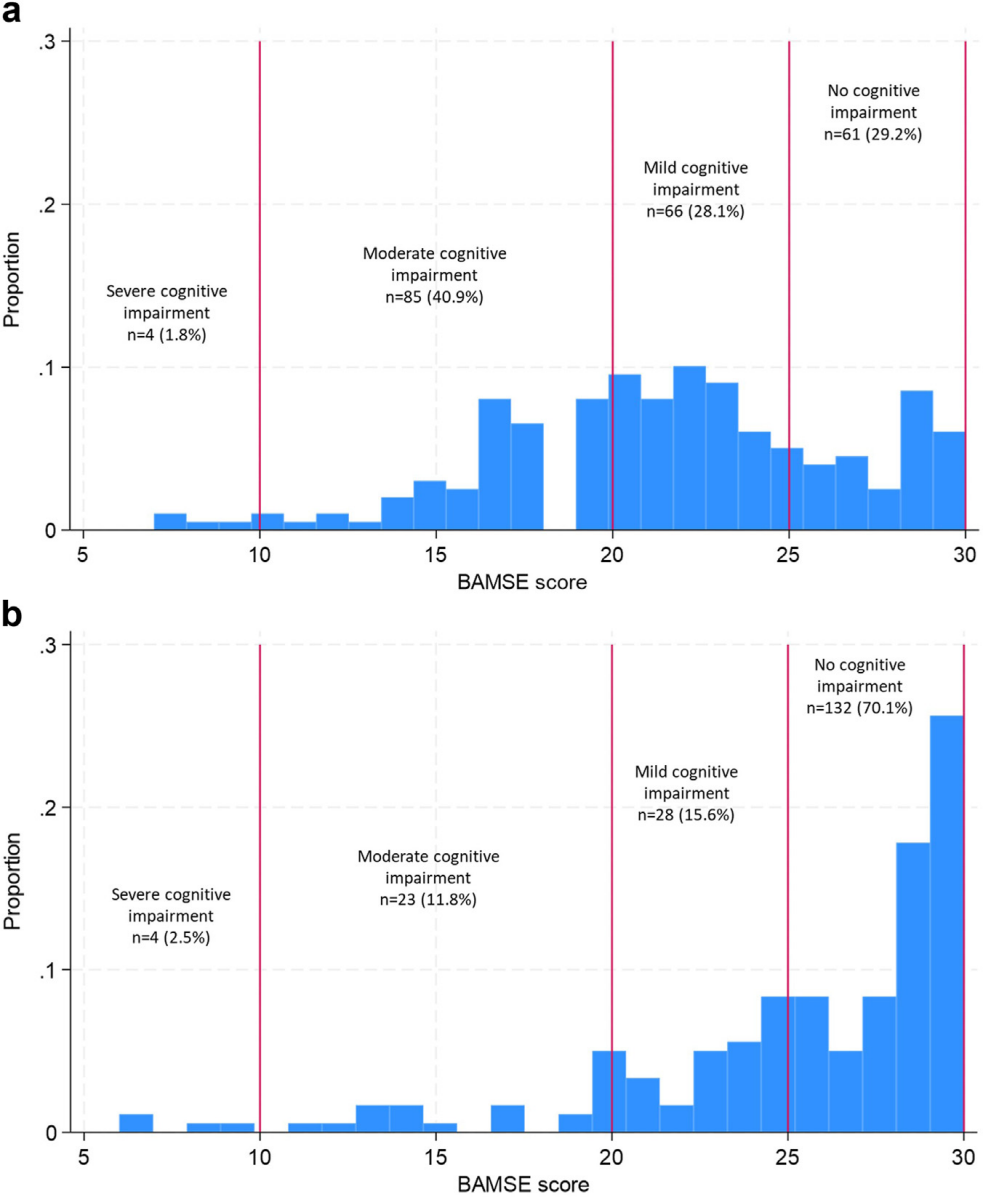
**a. Distribution of BAMSE scores among 216 female adults aged 60 years and above in Faridpur, with vertical lines indicating categories of cognitive impairment. b: Distribution of BAMSE scores among 187 male adults aged 60 years and above in Faridpur, with vertical lines indicating categories of cognitive impairment**.

Based on categorisations of different levels of cognitive impairment, more than 50% of the study population had at least mild cognitive impairment, with almost 1 in 3 experiencing moderate to severe impairment. Women (Fig. 2a) had lower scores (lower cognitive function) compared to men (Fig. 2b), with only 29.2% of women with no cognitive impairment compared to 70.1% of men.

**Fig. 3:**
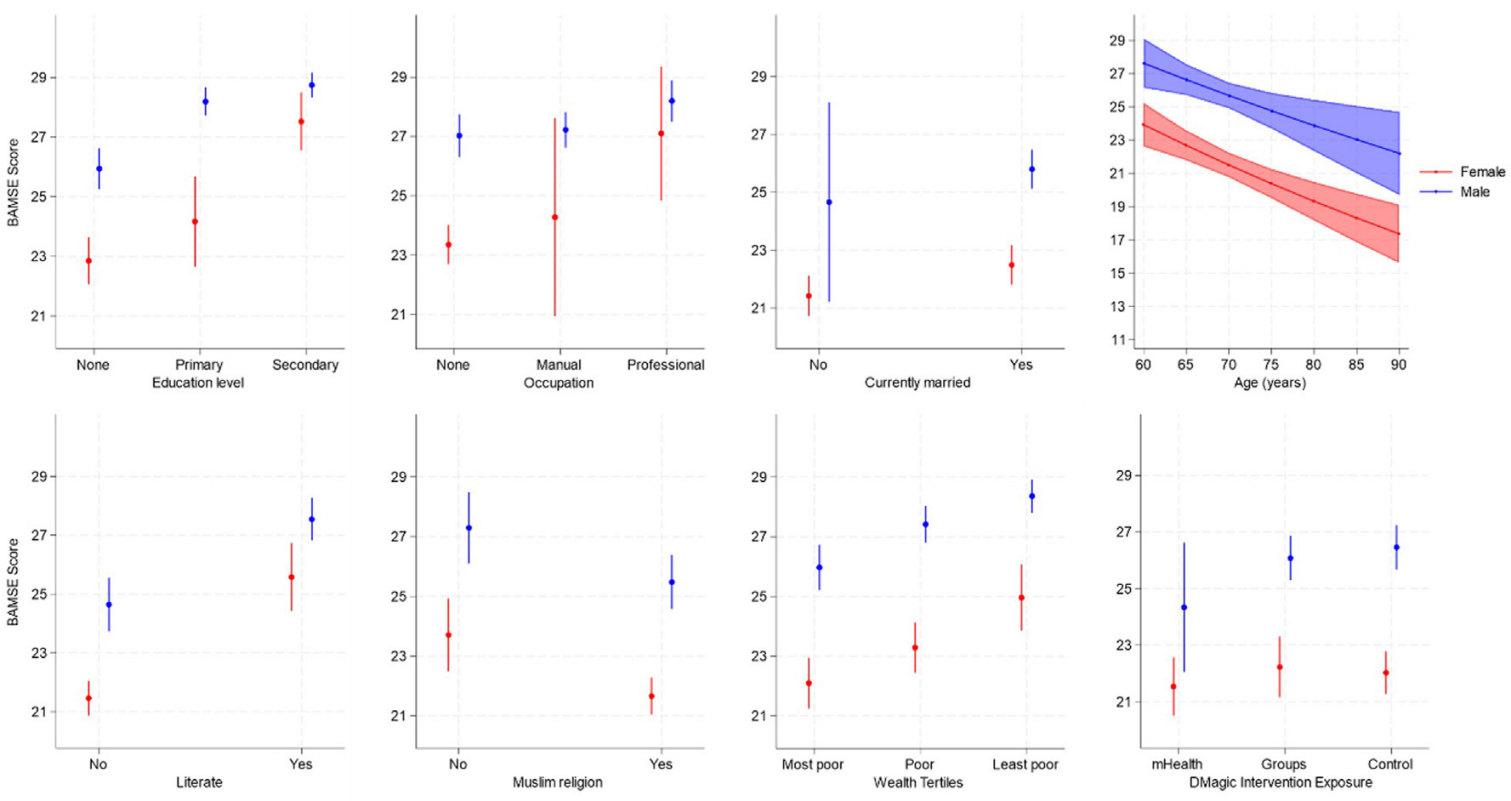
**Predicted marginal mean values of BAMSE (with 95% CI) by education level, occupation type, marital status, literacy, religion, wealth tertile and age for men and women separately in Faridpur, Bangladesh. Education, occupation, marital status, literacy and religion adjusted for age and wealth tertile. Wealth adjusted for age only. Age adjusted for wealth only**.

Increasing age was significantly associated with declining BAMSE score for men and women in crude analysis and when adjusted for wealth tertiles (Table 1). Among women, increasing education level, being in the least poor wealth tertile, ability to read, and being married were significantly associated with higher BAMSE score. Women engaged in manual labour had lower BAMSE scores compared to women with no paid employment, although small numbers are noted and there was no evidence of association when adjusted for age and wealth. The adjusted analysis suggests that women working in professional or clerical roles had higher BAMSE scores compared to women with no paid employment, but again small numbers should be noted. Among men, higher education, wealth and literacy were similarly associated with gains in BAMSE as observed among women. Effects of education and literacy appeared larger among women than men. There was no evidence of association between occupation and BAMSE among men. In contrast to the associations observed among women, there was no evidence of an association between being currently married and BAMSE among men. Being Muslim was significantly associated with lower BAMSE scores compared to other religions among men, but no significant association was observed among women. Among men, BAMSE was lower in villages exposed to the DMagic mHealth intervention, relative to control, but no evidence of association was observed among women. In contrast, the DMagic PLA intervention had a significant positive association with BAMSE among women, reflecting a relative 10% increase in BAMSE score. No association was observed between PLA exposure and BAMSE among men. Fig. 3 illustrates predicted marginal effects of socio-demographic factors on BAMSE scores for men and women based on the adjusted regression results.

Approximately one fifth of women had diabetes, 46.0% had hypertension and approximately 1 in 3 (31.2%) had BMI classified as overweight or obese (Table 2). The prevalence of diabetes was similar among men, though fewer men had hypertension (29.5%) or overweight/obesity (25.0%). Twenty-seven women (12.2%) were identified with moderate or mild depressive symptoms, according to PHQ-9 categorisations, and 23 (10.0%) had moderate or severe anxiety based on GAD-7 scores. Moderate or severe depressive symptoms and anxiety were less common among men, with a prevalence of 7.9% and 6.3% respectively.

**Table 2:** BAMSE score distributions and associations with socio-demographic and health parameters among adults aged 60 years and above in rural Faridpur, Bangladesh.

	Female				Male			
	N (weighted %) or mean (SD)	Median BAMSE score (IQR)	BAMSE score ratio (95% CI); p-value		N (weighted %) or mean (SD)	Median BAMSE score (IQR)	BAMSE score ratio (95% CI); p-value	
			Crude	Adjusted^a^			Crude	Adjusted^a^
Total	216 (100%)	22 (18–25)			187 (100)	26 (24–29)		
Diabetes								
No	175 (79.1%)	21 (18–25)	Ref	Ref	151 (81.4%)	26 (24–29)	Ref	Ref
Yes	41 (20.9%)	22 (20–25)	1.07 (0.99, 1.15); p = 0.097	1.03 (0.97, 1.10); p = 0.34	36 (18.6%)	28 (23–30)	1.00 (0.92, 1.09); p = 0.99	0.97 (0.89, 1.05); p = 0.41
Hypertension								
No	120 (54.0%)	22 (19–25)	Ref	Ref	129 (70.6%)	26 (23–29)	Ref	Ref
Yes	96 (46.0%)	21 (18–25)	0.98 (0.90, 1.05); p = 0.53	1.00 (0.94, 1.07); p = 0.91	58 (29.5%)	27 (24–30)	0.98 (0.91, 1.06); p = 0.62	0.97 (0.90, 1.04); p = 0.35
BMI, mean (SD)	21.5 (4.0)		1.02 (1.01, 1.03); p = 0.0001	1.01 (1.01, 1.02); p = 0.0002	21.0 (4.0)		1.01 (1.00, 1.02); p = 0.23	1.00 (0.99, 1.01); p = 0.70
Overweight/obesity								
No	145 (68.9%)	21 (18–24)	Ref	Ref	140 (75.0%)	26 (23–29)	Ref	Ref
Yes	71 (31.2%)	23 (20–27)	1.11 (1.02, 1.22); p = 0.020	1.10 (1.02, 1.19); p = 0.011	47 (25.0%)	29 (26–30)	1.07 (1.00, 1.14); p = 0.054	1.01 (0.94, 1.10); p = 0.70
Depressive symptoms								
None/mild	189 (87.9%)	22 (18–25)	Ref	Ref	173 (92.1%)	27 (24–29)	Ref	Ref
Moderate/Severe	27 (12.2%)	20 (15–23)	0.89 (0.77, 1.03); p = 0.12	0.94 (0.83, 1.06); p = 0.31	14 (7.9%)	23 (22–25)	0.87 (0.78, 0.98); p = 0.024	0.90 (0.82, 0.99); p = 0.037
Anxiety								
None/mild	193 (90.0%)	22 (18–25)	Ref	Ref	174 (93.7%)	26 (24–29)	Ref	Ref
Moderate/Severe	23 (10.0%)	20 (17–22)	0.89 (0.78, 1.02); p = 0.10	0.94 (0.82, 1.07); p = 0.36	13 (6.3%)	27 (23–29)	1.02 (0.93, 1.12); p = 0.68	1.02 (0.94, 1.10); p = 0.62
Current daily use of tobacco products								
No	79 (38.2%)	22 (20–26)	Ref	Ref	52 (26.3%)	26 (20–30)	Ref	Ref
Yes	137 (61.8%)	21 (17–25)	0.93 (0.87, 0.99); p = 0.018	0.94 (0.89, 1.00); p = 0.060	135 (73.7%)	26 (24–29)	1.08 (0.98, 1.18); p = 0.11	1.07 (0.99, 1.16); p = 0.086
At least 150 min of physical activity per week								
No	142 (62.9%)	20 (17–23)	Ref	Ref	113 (59.2%)	26 (23–29)	Ref	Ref
Yes	74 (37.1%)	24 (20–28)	1.14 (1.06, 1.23); p = 0.0007	1.08 (1.01, 1.16); p = 0.020	74 (40.8%)	27 (25–29)	1.05 (0.99, 1.11); p = 0.075	1.01 (0.95, 1.07); p = 0.76
Current daily consumption of betel								
No	76 (39.1%)	22 (20–28)	Ref	Ref	80 (41.6%)	26 (24–29)	Ref	Ref
Yes	140 (60.9%)	21 (18–24)	0.91 (0.85, 0.98); p = 0.011	0.94 (0.89, 1.00); p = 0.056	107 (58.4%)	27 (24–29)	1.01 (0.96, 1.08); p = 0.64	1.01 (0.96, 1.07); p = 0.61

a Adjusted for age, wealth tertile and DMagic intervention arm allocation.

**Fig. 4:**
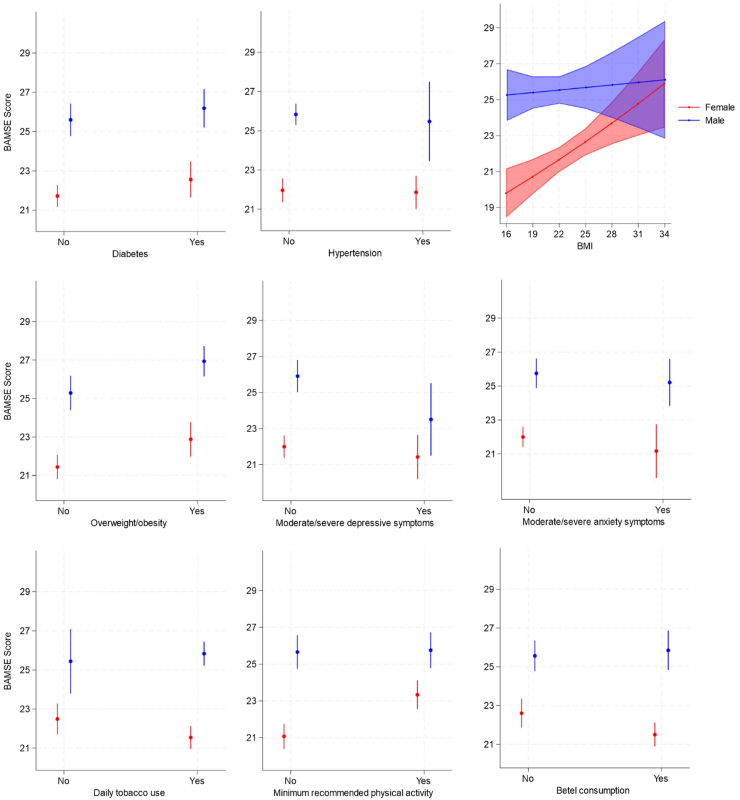
**Predicted marginal mean of BAMSE (with 95% CI) by health status and behavioural indicators on BAMSE for men and women separately in Faridpur, Bangladesh, adjusted for age, wealth tertile and DMagic intervention allocation**.

There was no evidence of association between diabetic status or hypertension and BAMSE among men or women (Table 2; Fig. 4). Results indicate a positive association between BMI and overweight/obesity and BAMSE among women. Moderate/severe depressive symptoms were associated with a decrease in BAMSE score, and significantly so among men.

61.8% of women and 73.7% of men reported current daily use of tobacco products. Among women who used tobacco, only 2 (1.6%) reported smoking, with all 137 (100%) reporting use of smokeless tobacco, including *zarda*, *sadapata* and *gul*. Approximately half of male tobacco consumers smoked (49.7%) and 72.7% reported use of smokeless tobacco products. Tobacco consumption was negatively associated with BAMSE among women; no association between tobacco use and BAMSE was observed among men (Table 2; Fig. 4).

37.1% of women reported physical activity levels that met the threshold of 150 min per week and those who met this threshold had a significantly higher BAMSE score compared to women who did not. The proportion of men meeting the threshold of at least 150 min of physical activity per week was slightly higher than that observed among women but, unlike women, there was no evidence of association between this behaviour and BAMSE score (Table 2; Fig. 4).

Almost 61% of women reported daily consumption of betel and this was associated with a significant decline in cognitive function as measured by the BAMSE tool (Table 2; Fig. 4). Betel consumption was slightly lower among men compared to women and there was no evidence of an association between its use and BAMSE in men.

## Discussion

We conducted a study of cognitive function in older adults in rural Bangladesh and explored the relationship with demographic, socioeconomic, and modifiable risk factors for dementia. We found that almost a third of all participants exhibited moderate to severe cognitive impairment and another 23% met criteria for mild cognitive impairment, with notably worse cognitive function scores among women than men. A previous smaller study of cognition in rural Bangladesh reported a prevalence of mild to moderate cognitive impairment in 21% of participants.^9^ Prevalence of dementia in rural Bangladesh has been estimated at 3.6% in persons aged over 60,^8^ lower than the reported standardised prevalence of 5.7% for South Asian countries.^33^ However, this estimate is based on one study to date that conducted structured clinical interviews of less than 500 older adults, of which a further 11.5% were classified as having a possible dementia diagnosis.^8^ The higher rates of cognitive impairment reported in our sample relative to other studies from Bangladesh or elsewhere may reflect sociodemographic differences, such as age, literacy and education levels, as well as variable definitions of cognitive impairment. For example, a recent study from India defined cognitive impairment as a score in the 10th centile^34^ which, if applied in our study would give prevalence of around 12% and 10% in women and men, respectively. Methodological differences notwithstanding, our findings indicate that a high proportion of this population require dementia assessment and exhibit a marked reduction in cognitive reserve placing them at significantly increased risk of developing dementia.

Our findings suggest that, for men and women, those at greatest risk of lower cognitive function were older, with less education, poor literacy and lower wealth. Among women, being married was associated with higher cognitive functioning (less impairment). We also identified notable sex differences in health and behaviours and associations with cognitive function. Current daily use of betel nut and daily consumption of tobacco were associated with worse cognitive function in women but not men, whereas higher physical activity was associated with higher cognitive function in women but not men. In men and women, our data suggest that overweight and obesity is associated with improved cognitive function in this population, though this is attenuated and non-significant upon adjustment in men.

We observed that, among men, residing in villages allocated to the mHealth intervention in the previous DMagic trial was associated with lower BAMSE score. The mHealth intervention entailed twice-weekly health behaviour and diabetes awareness-raising voice messages sent to participants’ mobile phones over a period of 14 months. The DMagic trial showed that this intervention raised population-level knowledge and awareness about diabetes^12^ but had no impact on disease outcomes or behaviours. Further, intervention effects on knowledge and awareness were no longer observed five years after randomisation.^13^ Subsequent equity analysis showed that the mHealth intervention reduced diabetes incidence among younger (30–40 year old) adults with intermediate hyperglycaemia prior to intervention.^35^ There is no clear mechanism by which the mHealth intervention or its effects might impact cognitive function in older adults and the association observed in the current study is likely to be anomalous or represent unmeasured differences between study clusters. The Participatory Learning and Action (PLA) DMagic intervention (a community mobilisation intervention that showed population-level impact on knowledge and hyperglycaemia), was associated with a significant 10% relative increase in BAMSE among women. Further research is needed to explore potential impacts of community mobilisation interventions such as PLA on cognitive function, and documented effects on diabetes^12^ and five-year hypertension risk^13^ offer plausible pathways to impact. All analyses of cognitive function associations with health and behavioural measures that could have plausibly been impacted by the DMagic interventions were controlled for intervention allocation.

The 2017 Lancet Dementia Commission^36^ concluded that a third of all dementia is potentially attributable to a combination of the following nine risk factors: education to a maximum of age 11–12 years, midlife hypertension, midlife obesity, hearing loss, late-life depression, diabetes, physical inactivity, smoking, and social isolation. However, these risk factors were largely based on epidemiological studies in western high-income settings and our findings highlight the common and contrasting factors that may drive cognitive health in these different populations.

Higher childhood education and lifelong educational attainment is associated with improved cognitive function and reduced dementia risk.^36^ Our findings support this evidence and other findings from Bangladesh,^7^^,^^8^ and identify improving education and literacy in rural Bangladesh as a potentially important intervention in preventing future cognitive decline, though this is unlikely to impact the current generation of older adults. Observed gender differences in educational attainment and in cognitive function highlight a need for increased efforts to improve access to education for girls in particular. Previously, more consistent evidence has supported this education–dementia relationship in high-compared to low-income countries.^37^ This is likely due to several factors including shorter life expectancy reducing dementia cases, access to education reflecting social class and wealth rather than intellectual promise or cognitive capacity, and other drivers of access to education including poorer maternal health, poor nutrition, or infection. Due to these factors and despite controlling for wealth, whether the association we observed between education and cognitive function leads to increased risk of dementia in those with no education and/or low literacy remains unclear. For example, a previous study in Mexico found that low education was associated with an increased risk for dementia in urban participants but not in rural participants.^38^ All interpretation of associations between education, literacy and cognitive function, however, should also recognise that cognitive testing assesses performance in abstract tasks, which may be less familiar to illiterate individuals and those who have received less education. As such, despite being adapted specifically for illiterate population, the BAMSE tool we used may still be subject to education and literacy bias.

Trials of physical activity in older adults have been shown to improve cognitive function,^39^ however the relationship between activity and risk of dementia is complex with the potential for both risk reduction and reverse causation.^36^ Our finding that increased physical activity in females is positively associated with cognition supports existing evidence of sex differences in the protective effect of physical activity on cognition and subsequent dementia risk.^40^ Due to the increased prevalence of dementia in women and gender gap in physical activity in low- and middle-income settings, studies have suggested that a reduction of 10% in physical inactivity only among women may reduce the total burden of dementia to the same extent as a 10% decrease in physical inactivity in the whole population in such settings.^40^ However, we did not observe a large sex difference in reported levels of physical activity in our sample. Another explanation is that physical activity has distinct neurobiological effects for men and women. A recent multimodal neuroimaging study of healthy older adults found sex-dependent associations between brain integrity and physical activity which may in turn drive risk of dementia.^41^ Reverse causality may also be a factor, whereby cognitive impairment makes it more difficult for individuals, and particularly women, to be physically active in settings such as rural Bangladesh where safety and harmful social norms are already barriers to female physical activity.^29^

Previous studies have shown that people who are lifelong single and those who are widowed have a 42% and 20% higher risk of developing dementia respectively than those who are married,^42^ and a recent meta-analysis also shows a significant association between widowhood and cognition.^43^ Similar to findings in West Bengal,^44^ being married may represent aspects of social contact in our study population. Social contact is an established protective factor in older adults that enhances cognitive reserve and reduces dementia risk.^36^ The positive effects of marriage may be explained by marital status changing individuals' exposure to other protective and risk factors throughout their lives, for example studies have shown that married people tend to be more likely to have a healthy lifestyle and have socioeconomic benefits,^42^ but social contact and the benefits this conveys in building cognitive reserve is also likely to be an important factor. Our data show important sex differences in marital status and in the association between being currently married and cognitive function among men and women.

Despite similar ages in men and women in our sample, the low proportion of elderly men who are not married likely reflects that female spouses typically outlive their husbands, husbands of elderly women are on average 10 years older than their wives, and there are high rates of remarriage for elderly men.^45^ Cultural norms associated with widowhood among women in South Asia have been found to cause interacting disadvantages such as inheritance refusal, constrained movement beyond the household, limited engagement in economic activities, prevention of remarriage, and restricted involvement in social activities.^46^^,^^47^ A study using a large nationwide cross-sectional survey in India showed that older widowed women in India have lower cognitive function compared to married women, even after controlling for socio-economic factors, and that physical and mental health are important mediators in the widowhood-cognition disparity.^48^ The results also showed that the negative impact of widowhood on cognition could persist for up to 20 years.

A qualitative study conducted in rural Bangladesh described the healthcare perceptions of 17 women aged 60 years or above, of which 11 were widowed.^49^ The study revealed a core theme of social exclusion. This exclusion encompasses inferior roles, restricted access to resources, and mobility limitations. Decision-making, often controlled by husbands or, in their absence, the eldest son, contributes to healthcare disparities. Economic barriers arise from harmful gender roles, limiting women's access to means of production. Poverty and medication costs further restrict healthcare seeking and the health of older women is usually the least prioritised within the family. Cultural practices, like purdah, impose mobility restrictions and require escorts for healthcare visits. There is a need for further research to understand the mechanisms by which widowhood might be associated with poorer cognition among women, to understand the social, economic, and religious factors that influences this, and to design interventions specifically for those who have experienced spousal bereavement.^43^

Our finding that BMI and overweight and obesity are associated with improved cognition among women and a lack of association in the adjusted model between cardiometabolic risk factors such as hypertension and diabetes and cognitive function appears counterintuitive given existing evidence. Midlife obesity is associated with increased dementia risk and worse cognitive function which weight loss can reverse to a degree, while hypertension and diabetes are consistently associated with poorer cognition and increased dementia risk.^50^ However, the relationship is more complex, the largest meta-analysis of longitudinal studies to date found that obesity but not being overweight was associated with late-life dementia.^51^ Studies in low- and middle-income countries including Bangladesh have also found that malnutrition, rather than being overweight, is a key driver of dementia risk^8^ and cognitive function^52^ and another study in India replicated our finding showing a clear pattern of higher weight and improved cognition.^40^ In addition, cardiometabolic risk factors for cognitive impairment and dementia vary by country. A study of HbA1c level, a measure of long-term glycaemic control, found that higher levels were associated with cognitive impairment in older adults in the USA but improved cognitive function in older adults in India.^40^ This may reflect the different relationship between socioeconomic status and nutrition in high-income countries compared to rural low- and middle-income populations where higher weight may be associated with other socioeconomic factors such as wealth and education. The possibility for reverse-causality in our observed BMI-cognition association may also be possible, whereby those with worse cognition lose weight through changed eating habits.

Our finding that depressive symptoms were associated with lower BAMSE score in men supports substantial existing evidence that depression is associated with cognitive impairment and increased risk of dementia. However, a previous study in France examining the gender specific effects of depression on mild cognitive impairment and dementia reported depressive symptoms were associated with women, not men, highlighting the need for further study of gender specific cognitive risk factors in different populations.^53^ Debate remains as to whether depression is an early symptom or risk factor for dementia.^54^ A diagnosis of depression is associated with a two-fold increase in risk of dementia, and greater number of depressive episodes increase this risk.^55^ However, in cognitively normal older adults, biomarkers of Alzheimer's disease pathology are associated with subsequent depression,^56^ supporting the hypothesis that depression is an early prodromal symptom of dementia. These explanations are not mutually exclusive and longitudinal studies have highlighted the timing of depression as an important factor in defining the nature of the association between depression and dementia.^57^

Tobacco use is an established risk factor for dementia and poorer cognition.^58^ Although our results indicate an association between tobacco use and worse cognition in women, this effect was not observed for men. This finding may be a consequence of the complex and poorly understood effects of varying types and pattern of tobacco use on later-life cognitive health in rural low-income settings. Modes of tobacco consumption vary between men and women and differing effects of smoked and chewed tobacco on cognitive impairment may be important to investigate.^59^

Betel nut use is the fourth most commonly used psychostimulant (after caffeine, alcohol, and nicotine) globally with over 600 million users.^60^ Despite this widespread use few studies have examined the association with cognition. A previous study in Taiwan reported no association between betel nut use and cognition in older adults,^61^ however, betel-nut chewing is associated with increased metabolic disease, cardiovascular disease, and all-cause mortality.^62^ Neuroimaging studies^63^^,^^64^ have reported brain alterations in structure, metabolism and function in betel nut users and insights into the pathophysiological mechanisms of memory disorders in betel nut users are emerging.^65^ Our findings suggest potentially harmful cognitive effects in women highlighting the need for further study of the gender specific cognitive effects of long-term betel nut use as well as cultural and social drivers of this risk behaviour.

### Strengths and limitations

Our study reports a random population-based sample of cognitive health and its predictors in Bangladesh that combines both objective (blood pressure, BMI, blood glucose) and locally adapted (BAMSE, PHQ and GAD) measures to explore the relationships of previously established risk factors for cognitive impairment in this population. Data collection using professionals with a health background may help to reduce measurement error in relation to cognitive and other health outcomes. This was not feasible in our context and so we placed great importance on fieldworker training, supervision and data quality control to minimise measurement error.

Our sample is restricted to a rural population in just one district of Bangladesh and our response rates indicate that sampled adults with higher ages were less likely to participate. As a sub-group analysis of a larger survey, sample size is relatively small in some categories, with implications for statistical power. Being mindful of this, and of the multiple statistical tests conducted, our reporting and interpretation of results focuses on direction of association and confidence intervals, rather than thresholds of statistical significance.

There are also important dimensions that were not measured within our study. We did not clinically assess participants therefore pathological causes of cognitive impairment in participants including dementia, endocrinological disorders such as thyroid problems or vitamin B12 deficiency are unknown. Similarly, we did not assess other aspects of functional health associated with cognitive function, such as hearing loss. Although we were able to use marriage and occupation as indirect measures, we did not directly measure number of social connections. Many of our measures were also reliant on self-report and therefore susceptible to bias.

In the context of an analysis to describe distributions and associations with cognitive function, there can be uncertainty over which highly correlated variables to adjust for. Our analysis adjusts for age and wealth, which we consider to be critical factors that correlate with BAMSE and with most socio-economic parameters. Nevertheless, results should be interpreted with consideration of potential residual confounding and effects of unmeasured parameters.

Finally, our cross-sectional study design prevents causal inference on the associations we observed and extensive sub-group analysis is limited by sample size.

### Conclusion

A high proportion of older adults in rural Bangladesh exhibit cognitive impairment placing them at increased risk of dementia. Developing and investing in health and social care systems in this region with clear diagnostic, treatment and support strategies for individuals with cognitive impairment and their caregivers are needed. Considering notable gender difference, gender specific interventions may be needed across the lifecourse^66^ to improve cognition in this population, including physical activity for women and reducing social isolation. Effective behaviour change interventions will require recognition of cultural and social barriers to healthy behaviours. However, further longitudinal research is needed to understand the cognitive trajectory of individuals and establish any causal role between health behaviours and cognitive function in this low-income rural population.

## Contributors

EF was Principal Investigator of the DMagic and DClare studies, co-designed the current study and analysis, conducted data analysis and wrote the first draft of the Methods and Results. HC co-designed the current study, wrote the first draft of the Introduction and Discussion and contributed significantly to the interpretation of study findings. AK, NA, SKS, TN, CK, HHB, MP, AC, JM and KA designed and implemented survey methods, data collection and data processing, contributed to interpretation of results and contributed to revisions of the draft manuscript. KA was co-Principal Investigator of the DMagic and DClare studies. EF & CK had access to and verified the data. All authors were jointly were responsible for the decision to submit the manuscript.

## Data sharing statement

De-identified data collected for this study and a data dictionary are available from the corresponding author on reasonable request.

## Declaration of interests

Recipients of funding for this work were Principal Investigator Fottrell and Co-investigators King, Copas, Azad, Khan, Kuddus, Haghparast-Bidgoli, and Morrison. Co-authors Ahmed, Shaha, Pires and Nahar were employed on the project using project funding. Costello did not receive any funding for this work.

The authors declare no competing interests.

## Appendix A Supplementary data

Translate abstract
