# Supplementary material for: Cognitive function and associations with demographic, socioeconomic, health and behavioural factors among older adult men and women in rural Bangladesh: a population-based cross-sectional study
Source: Lancet Reg Health Southeast Asia. 2025 Apr 7;36:100575. doi: 10.1016/j.lansea.2025.100575 (PMC12004372; doi:10.1016/j.lansea.2025.100575)
Supplement: Translate abstract [file mmc1.docx]

**Editor’s Note:** this translated abstract has not been peer-reviewed.

**সারাংশ**

**পটভূমি**
জ্ঞানগত বা জ্ঞানীয় অক্ষমতা স্বাস্থ্য, জীবনমান এবং বেঁচে থাকার উপর গুরুত্বপূর্ণ প্রভাব ফেলে এবং এর বাড়তি বোঝা বিশ্বব্যাপী একটি গুরুতর স্বাস্থ্য চ্যালেঞ্জ তৈরি করে। নিম্ন-সম্পদ সম্পন্ন অঞ্চলের বয়স্কদের মধ্যে জ্ঞানগত কার্যকারিতা এবং এর সম্পর্কিত জনসংখ্যাগত, সামাজিক-অর্থনৈতিক, স্বাস্থ্য এবং আচরণগত কারণগুলির উপর ভিত্তি করে অভিজ্ঞতামূলক জনসংখ্যাভিত্তিক গবেষণাগুলি খুবই কম। এই গবেষণাটি বাংলাদেশের গ্রামীণ এলাকায় বয়স্কদের মধ্যে জ্ঞানগত অক্ষমতার বোঝা এবং জনসংখ্যাগত, স্বাস্থ্য এবং আচরণগত কারণগুলির সাথে এর সম্পর্ক বর্ণনা করে।

**পদ্ধতি**
বাংলাদেশের ৯৬টি গ্রামে ৬০ বছর বা তার বেশি বয়সী পুরুষ এবং মহিলাদের মধ্যে একটি দৈবচয়ন নমুনায় জনসংখ্যাভিত্তিক একক-অবস্থানে গবেষণা পরিচালনা করা হয়। বাংলা অ্যাডাপ্টেশন অফ দ্য মিনি-মেন্টাল স্টেট এক্সামিনেশন (BAMSE) ব্যবহার করে জ্ঞানীয় কার্যকারিতা পরিমাপ করা হয়েছিল, যেখানে উচ্চতর স্কোর উচ্চতর কার্যকারিতা নির্দেশ করে। স্ট্যান্ডার্ড প্রোটোকল ব্যবহার করে রক্তচাপ, উচ্চতা এবং ওজন পরিমাপ করা হয়েছিল এবং ডায়াবেটিসের ঝুঁকি সনাক্ত করতে ওজিটিটি দ্বারা অভুক্ত অবস্থায় এবং গ্লুকোজ খাওয়ার ২-ঘন্টা পর পরীক্ষা করা হয়েছিল। সাক্ষাৎকার গ্রহণকারী প্রশ্নাবলীর মাধ্যমে বিষণ্নতার লক্ষণ, উদ্বেগ এবং আত্ম-প্রতিবেদিত স্বাস্থ্য আচরণ মূল্যায়ন করা হয়। পুরুষ ও মহিলাদের জন্য আলাদাভাবে বিশ্লেষণ করা হয়েছিল এবং শক্তিশালী পয়সন রিগ্রেশন ব্যবহার করে BAMSE স্কোরের সাথে সমাজ-জনসংখ্যাগত, স্বাস্থ্য এবং আচরণগত কারণগুলির মধ্যে সম্পর্ক পরীক্ষা করা হয়েছিল।

**ফলাফল**
৪০৩ জন (২১৬ জন মহিলা, ১৮৭ জন পুরুষ) যোগ্য অংশগ্রহণকারীর কাছ থেকে তথ্য সংগ্রহ করা হয়। ৫০% এর বেশি জনগণের কমপক্ষে মৃদু জ্ঞানগত অক্ষমতা ছিল এবং মহিলাদের জ্ঞানগত স্কোর পুরুষদের তুলনায় কম ছিল। তরুণ বয়স, উচ্চ শিক্ষা, ধনসম্পদ এবং সাক্ষরতা পুরুষ ও মহিলাদের মধ্যে BAMSE স্কোরের সাথে উল্লেখযোগ্যভাবে সম্পর্কিত ছিল। বৈবাহিক অবস্থার সাথে পুরুষ ও মহিলাদের মধ্যে সম্পর্ক ভিন্ন ছিল, যেখানে মহিলাদের মধ্যে বিবাহিত হওয়া BAMSE-এর সাথে একটি ইতিবাচক সম্পর্ক ছিল, অর্থাৎ উচ্চতর জ্ঞানগত কার্যকারিতা {আপেক্ষিক স্কোর অনুপাত (রেলাটিভ স্কোর

রেশিও) (৯৫% CI) ১.০৮ (১.০২, ১.১৫), p=০.০১৩), তবে পুরুষদের মধ্যে কোনো সম্পর্ক ছিল না (০.৯৪ (০.৮৭, ১.০২), p=০.১৩}। ডায়াবেটিস বা উচ্চ রক্তচাপের সাথে কোনো স্পষ্ট সম্পর্ক পর্যবেক্ষণ করা হয়নি, তবে অতিরিক্ত ওজন এবং স্থূলতা মহিলাদের মধ্যে BAMSE স্কোর বাড়ানোর সাথে সম্পর্কিত ছিল (১.১০ (১.০২, ১.১৯), p=০.০১১), কিন্তু পুরুষদের মধ্যে নয় (১.০১ (০.৯৪, ১.১০), p=০.৭০)। মাঝারি এবং গুরুতর বিষণ্নতার লক্ষণ পুরুষদের মধ্যে কম BAMSE স্কোরের সাথে সম্পর্কিত ছিল (০.৯০ (০.৮২, ০.৯৯), p=০.০৩৭), কিন্তু মহিলাদের মধ্যে নয় (০.৯৪ (০.৮৩, ১.০৬), p=০.৩১)। শারীরিক কার্যকলাপ মহিলাদের মধ্যে BAMSE স্কোরের আপেক্ষিক বৃদ্ধির সাথে সম্পর্কিত ছিল (১.০৮ (১.০১, ১.১৬), p=০.০২০), তবে পুরুষদের মধ্যে কোনো সম্পর্ক দেখা যায়নি (১.০১ (০.৯৫, ১.০৭), p=০.৭৬)। পানের সেবন মহিলাদের মধ্যে BAMSE স্কোর কমানোর সাথে সম্পর্কিত ছিল (০.৯৪ (০.৮৯, ১.০০), p=০.০৫৬), কিন্তু পুরুষদের মধ্যে কোনো সম্পর্ক দেখা যায়নি (১.০১ (০.৯৬, ১.০৭), p=০.৬১)।

**ব্যাখ্যা**
বাংলাদেশের গ্রামীণ অঞ্চলের বড় একটি অংশের বয়স্ক জনগণের মধ্যে জ্ঞানগত বা জ্ঞানীয় কার্যকারিতা ব্যাহত হয়েছে এবং এই জনসংখ্যার মধ্যে জ্ঞানগত স্কোর এবং এর সম্পর্কিত জনসংখ্যাগত, সামাজিক-অর্থনৈতিক, স্বাস্থ্য এবং আচরণগত কারণে গুরুত্বপূর্ণ লিঙ্গভিত্তিক পার্থক্য রয়েছে। স্বাস্থ্য এবং সামাজিক সেবার সিস্টেম উন্নত করার জন্য, এই প্রেক্ষাপটের বিশেষ সামাজিক, অর্থনৈতিক এবং লিঙ্গভিত্তিক দৃষ্টিকোণগুলি বিবেচনায় নিয়ে জ্ঞানগত অবনতি প্রতিরোধ এবং ব্যবস্থাপনা করতে হবে।

**অর্থায়ন**
এই জরিপ এবং বিশ্লেষণটি বাংলাদেশের ডায়াবেটিস কমিউনিটি-লেড অ্যাওয়ারনেস, রেসপন্স অ্যান্ড ইভ্যালুয়েশন (DClare) গবেষণার একটি অংশ, যা UKRI/MRC (MR/T023562/1) দ্বারা গ্লোবাল অ্যালায়েন্স ফর ক্রনিক ডিজিজেস স্কেল-আপ প্রোগ্রামের অধীনে অর্থায়ন করা হয়েছে।

**Abstract**

**Background**

Cognitive impairment has a major impact on health, quality of life and survival and its increasing burden presents a critical global health challenge. Empirical population-based studies of cognitive function and its association with demographic, socioeconomic, health and behavioural factors among older adults in low-resource setting are rare. This study describes the burden of cognitive impairment and associations with demographic, health and behavioural factors among older adults in rural Bangladesh.

**Methods**

We conducted a population-based cross-sectional study of a random sample of men and women aged 60 years and above in 96 rural villages in Bangladesh. Cognitive function was measured using the Bangla Adaptation of the Mini-mental State Examination (BAMSE), where higher score indicates higher function. Blood pressure, height and weight were measured using standard protocols and fasting glucose and 2-hour oral glucose test were used to identify diabetes risk. Interviewer administered survey questionnaires assessed depressive symptoms, anxiety and self-reported health behaviours. Analyses were carried out separately for men and women and examined associations between sociodemographic, health and behaviour factors with BAMSE scores using robust Poisson regression.

**Findings**

Data were gathered from 403 (216 female, 187 male) eligible participants. More than 50% of the population had at least mild cognitive impairment and women had lower cognitive scores than men. Younger age, higher education, wealth, and literacy were significantly associated with higher BAMSE scores among women and men. Associations with marital status varied between men and women, with being married having a positive association with BAMSE among women, i.e. higher cognitive function (relative score ratio (95% CI) 1.08 (1.02, 1.15), p=0.013), but no association among men (0.94 (0.87, 1.02), p=0.13). No clear associations were observed with diabetes or hypertension, but overweight and obesity were associated with an increased BAMSE score among women (1.10 (1.02, 1.19), p=0.011) but not men (1.01 (0.94, 1.10), p=0.70). Moderate and severe depressive symptoms were associated with lower BAMSE scores among men (0.90 (0.82, 0.99), p=0.037), but not women (0.94 (0.83, 1.06), p=0.31). Physical activity was associated with a relative increase BAMSE score (1.08 (1.01, 1.16), p=0.020) among women, though no association was seen in men (1.01 (0.95, 1.07), p=0.76). The consumption of betel was associated with lower BAMSE among women (0.94 (0.89, 1.00), p=0.056), but there was no evidence of association among men (1.01 (0.96, 1.07), p=0.61).

**Interpretation**

A large proportion of older adults in rural Bangladesh have impaired cognitive function and there are important gender differences in the distribution of cognitive scores and their association with demographic, socioeconomic, health and behavioural factors in this population. Improvement in health and social care systems, taking into account the specific social, economic and gender dimensions of the context, are needed to prevent and manage the burden of cognitive decline in rural Bangladesh.

**Funding**

This survey and analysis were part of the Bangladesh Diabetes Community-Led Awareness, Response and Evaluation (DClare) study funded by UKRI/MRC (MR/T023562/1) under the Global Alliance for Chronic Diseases Scale-Up Programme.
